# Barley Yellow Mosaic Virus VPg Is the Determinant Protein for Breaking eIF4E-Mediated Recessive Resistance in Barley Plants

**DOI:** 10.3389/fpls.2016.01449

**Published:** 2016-09-30

**Authors:** Huangai Li, Hideki Kondo, Thomas Kühne, Yukio Shirako

**Affiliations:** ^1^Graduate School of Agricultural and Life Sciences, The University of TokyoTokyo, Japan; ^2^Asian Natural Environmental Science Center, The University of TokyoTokyo, Japan; ^3^Institute of Plant Science and Resources, Okayama UniversityKurashiki, Japan; ^4^Institute for Epidemiology and Pathogen Diagnostics, Julius Kühn-InstitutQuedlinburg, Germany

**Keywords:** barley yellow mosaic virus, eIF4E, *rym4*/*5*/*6*, resistance-breaking, VPg, virus replication, virus movement, mesophyll protoplast

## Abstract

In this study, we investigated the barley yellow mosaic virus (BaYMV, genus *Bymovirus*) factor(s) responsible for breaking eIF4E-mediated recessive resistance genes (*rym4*/*5*/*6*) in barley. Genome mapping analysis using chimeric infectious cDNA clones between *rym5*-breaking (JT10) and *rym5*-non-breaking (JK05) isolates indicated that genome-linked viral protein (VPg) is the determinant protein for breaking the *rym5* resistance. Likewise, VPg is also responsible for overcoming the resistances of *rym4* and *rym6* alleles. Mutational analysis identified that amino acids Ser-118, Thr-120, and His-142 in JT10 VPg are the most critical residues for overcoming *rym5* resistance in protoplasts. Moreover, the *rym5*-non-breaking JK05 could accumulate in the *rym5* protoplasts when eIF4E derived from a susceptible barley cultivar was expressed from the viral genome. Thus, the compatibility between VPg and host eIF4E determines the ability of BaYMV to infect barley plants.

## Introduction

Viral diseases cause serious economic losses by reducing both the quality and quantity of crop production (Kang et al., 2005a). The breeding of resistant crop plants is the most acceptable and applicable approach to control viral diseases because it is cost effective in terms of labor and material resources and, most importantly, it has no negative impact on the environment, with the exception of imposing strong selective pressure on virus populations (Clay and Kover, 1996; Kang et al., 2005a; Zhu et al., 2012). Of all known resistances against plant viruses, a large fraction is recessively inherited, with the majority of genes encoding for eukaryotic translation initiation factors eIF4E, eIF4G, or their isoforms (Fraile and García-Arenal, 2012; Wang and Krishnaswamy, 2012; Sanfaçon, 2015).

The eIF4E-mediated resistances in crop plants confer qualitative, genotype-specific resistance to viruses in the genus *Potyvirus* of the family *Potyviridae* (Kyle and Palloix, 1997; Ruffel et al., 2002, 2005, 2006; Nicaise et al., 2003; Gao et al., 2004; Kang et al., 2005a; Bruun-Rasmussen et al., 2007; Roudet-Tavert et al., 2007; Charron et al., 2008; Andrade et al., 2009; Naderpour et al., 2010) and occur through amino acid substitutions in eIF4E proteins encoded by the alleles (Ruffel et al., 2006; Yeam et al., 2007). The transient expression of eIF4E from susceptible cultivars renders the resistant cultivars to be susceptible to a certain virus pathotype (Ruffel et al., 2002, 2006). eIF4E in eukaryotic cells is an essential translation initiation factor that recruits the small ribosomal subunit (40S) to the mRNA cap structure, an event which is considered the first step in cap-dependent translation initiation (Malys and McCarthy, 2011). Moreover, it has been hypothesized that eIF4E also plays multiple roles in diverse processes during potyvirus infection, including translation, replication and cell-to-cell movement (Robaglia and Caranta, 2006; Truniger and Aranda, 2009). However, the precise roles of eIF4E during virus infection have yet to be demonstrated.

The viruses in the family *Potyviridae* contain a monopartite or bipartite (in the genus *Bymovirus*) positive-sense RNA genome that has a genome-linked viral protein (VPg) at the 5′ end and is polyadenylated at the 3′ end (Adams et al., 2012). The genome encodes one or two polyproteins that produce 10 mature proteins by self-encoded proteinases and P3N-PIPO (pretty interesting *Potyviridae* ORF) protein, a frameshift product from P3 (third protein) via a polymerase slippage (Adams et al., 2005, 2012; Chung et al., 2008; Olspert et al., 2015; Rodamilans et al., 2015). eIF4E-mediated resistance against potyviruses is overcome largely by viral VPg (Truniger and Aranda, 2009) and, to a lesser extent, P1 (first protein/protease; Nakahara et al., 2010), P3 (Hjulsager et al., 2006), CI (cytoplasmic inclusion protein; Abdul-Razzak et al., 2009; Tavert-Roudet et al., 2012), and probably HC-Pro (helper component protease; Ala-Poikela et al., 2011). The interaction between the host eIF4E and VPg is required for viral infection, and the interaction between these two proteins has been shown for several potyviruses (Léonard et al., 2000; Schaad et al., 2000; Kang et al., 2005b; Beauchemin et al., 2007; Roudet-Tavert et al., 2007; Yeam et al., 2007; Charron et al., 2008; Gallois et al., 2010; Mazier et al., 2011; Estevan et al., 2014).

Barley yellow mosaic virus (BaYMV), which belongs to the genus *Bymovirus* in the family *Potyviridae*, is one of the two causal agents [the other is barley mild mosaic virus (BaMMV)] of the economically important yellow mosaic disease of winter barley (*Hordeum vulgare* L.) in Europe and East Asia. In addition to their bipartite RNA genome, another feature of bymoviruses that distinguishes them from the members of other genera in the family *Potyviridae* is their transmission in soil by the root-inhabiting vector *Polymyxa graminis* Ledingham, a plasmodiophoraceous parasite (Adams et al., 2012; Tamada and Kondo, 2013). Like other plasmodiophorid-transmitted viral diseases, the planting of virus-resistant cultivars is a common available way to control yellow mosaic diseases (Ordon et al., 2009). Currently, a total of 18 resistance genes (15 recessive *rym* genes and three dominant *Rym* genes) against BaYMV (and also BaMMV) have been identified in barley, of which *rym4, rym5*, and *rym6* (*rym4*/*5*/*6*) resistances are recognized as allelic genes encoding for eIF4E on a region of barley chromosome 3H (Kanyuka et al., 2005; Stein et al., 2005). The emergence of a virulent bymovirus in resistant barley varieties (mainly in *rym4*/*5*/*6*-carrying cultivars) potentially causes a serious threat to barley production (Kühne, 2009). The *rym5* resistance gene, which was originally used in Japanese barley breeding programs (Kobayashi et al., 1987; Konishi and Kaiser, 1991), was overcome by a resistance-breaking isolate (Kashiwazaki et al., 1989). Moreover, *rym6*-mediated resistance also was overcome by this or other virulent isolates (Sotome et al., 2010; You and Shirako, 2013). Similarly, the *rym4* and, more recently, *rym5* genes, which are the major source of resistance for barley varieties in European countries, have been overcome by virulent BaYMV and BaMMV isolates (Hariri et al., 2003; Habekuss et al., 2008; Kühne, 2009). By sequence comparison between resistance-breaking and resistance-non-breaking isolates, VPg is suggested to be a determinant bymoviral protein responsible for breaking barley eIF4E-mediated resistance (Kühne et al., 2003; Kanyuka et al., 2004; Habekuss et al., 2008; Nishigawa et al., 2008). In addition, both the viral VPg and the host eIF4E are involved in host cell tropism (barley or wheat) of bymoviruses (Li and Shirako, 2015).

In this study, we utilized infectious cDNA clones derived from the resistance-breaking and resistance-non-breaking isolates of BaYMV to investigate the mechanisms underlying *rym4*/*5*/*6* eIF4E-mediated resistance in barley plants. Our results identified VPg as the determinant protein for breaking of *rym4*/*5*/*6* resistances. Moreover, mutational analyses combined with protoplast and whole plant inoculation assays suggest that the genetic compatibility between virally encoded VPg and host eIF4E regulates BaYMV infection at both the intracellular and intercellular (cell-to-cell and/or long-distance movement) levels.

## Materials and Methods

### Plants

Ryofu seeds were purchased from a local Japan Agriculture Cooperative branch. Express barley (carrying *rym4*) and Maris Otter seeds were from the Institute for Epidemiology and Pathogen Diagnostics, Germany. Other barley (carrying *rym5*: Mikamo Golden, Misato Golden; carrying *rym6*: Haruna Nijo, Amagi Nijo, and Miho Golden; KoA) seeds were obtained from the Institute of Plant Science and Resources at Okayama University with the support in part by the National Bio-Resource Project of the Ministry of Education, Culture, Sports, Science and Technology (MEXT), Japan.

### Virus Purification and Sequence Determination

BaYMV virions were purified from 2 g of infected leaves by a procedure described previously (Shirako and Brakke, 1984) and suspended in RNase-free H_2_O. Virus suspension was treated with Proteinase K at 37°C for 30 min, and viral RNA was extracted for cDNA synthesis. First-strand cDNA was synthesized using PrimeScript^®^ Reverse Transcriptase (Takara Bio, Japan) and then amplified by overlapping RT-PCR using PrimeSTAR^®^ HS DNA Polymerase (Takara Bio, Japan). All amplification products were purified using the FastGene^TM^ Gel/PCR Extraction Kit (Nippon Genetics, Japan) and then directly submitted to a commercial sequencing service (Eurofins Operon, Japan).

### Construction of Full-Length cDNA Clones of BaYMV Tochigi Isolate JT10 RNA1 and RNA2

**FIGURE 1:**
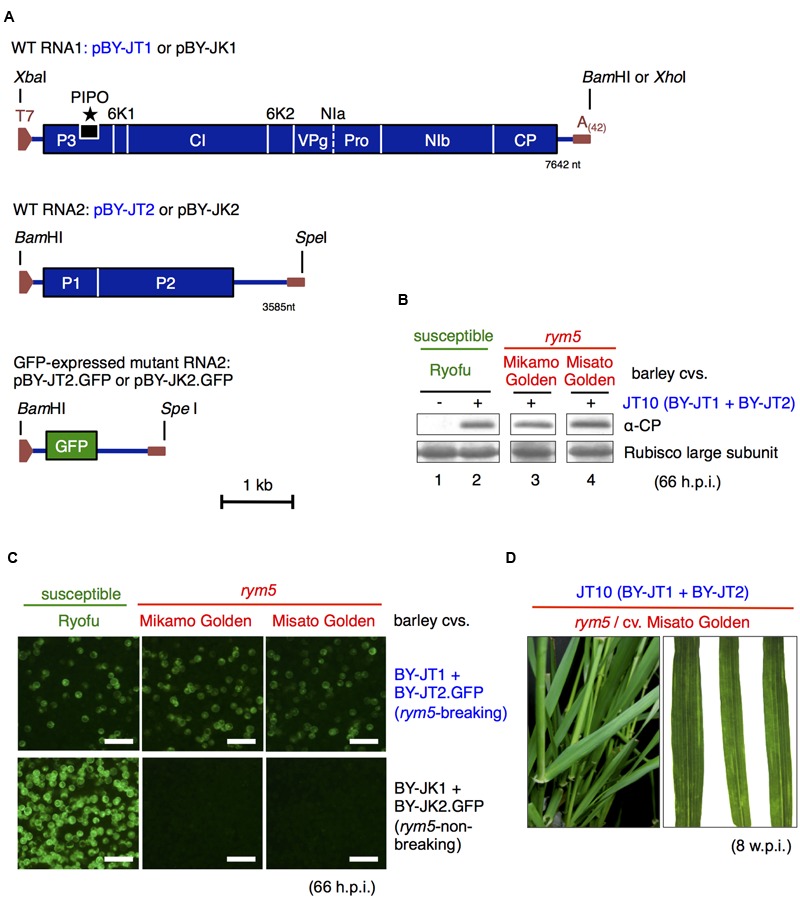
**Infectivity of *in vitro* transcripts derived from barley yellow mosaic virus JT10 cDNA clones. (A)** Schematic representation of full-length cDNA clones of JT10 (*rym5*-breaking) isolate (pBY-JT1 and pBY-JT2) and JK05 (*rym5*-non-breaking) isolate (pBY-JK1 and pBY-JK2). pBY-JT2.GFP and pBY-JK2.GFP are green fluorescent protein (GFP)-expressing RNA2 constructs generated by replacing P1 and P2 coding regions with the *GFP* gene. **(B)** Western blot analysis of coat protein (CP) accumulation in susceptible and *rym5* protoplasts transfected with JT10 *in vitro* transcripts. The rubisco large subunits were stained by Coomassie Brilliant Blue G-250 and used as a loading control. **(C)** GFP fluorescence in susceptible and *rym5* protoplasts transfected with *in vitro* transcripts of JT10 or JK05 RNA1 (pBY-JT1 or pBY-JK1) and GFP-expressing RNA2 (pBY-JT2.GFP or pBY-JK2.GFP). Bars, 200 μm. **(D)** Viral symptoms in upper systemic leaves of *rym5* plants following inoculation with JT10 *in vitro* transcripts. h.p.i., hours post-inoculation; w.p.i., weeks post-inoculation.

We have isolated a *rym5*-breaking BaYMV isolate (named isolate JT10) from diseased leaves of a *rym5*-carrying barley cultivar (Mikamo Golden) in Tochigi Prefecture, Japan in 2010 (Shirako and Li, unpublished data). Construction of infectious full-length cDNA clones of Tochigi isolate JT10 RNA1 (encoding polyprotein 1, P1) and RNA2 (encoding polyprotein 2, P2) were performed as described for Kurashiki isolate JK05 (You and Shirako, 2010). To generate a full-length cDNA clone of JT10 RNA1, named pBY-JT1, we performed RT-PCR using the TB162 primer for first-strand cDNA synthesis and TB87 and TB162 primers for the PCR procedure. The amplified 7.8-kb product was purified and digested with *Xba*I and *Bam*HI and then cloned into the *Xba*I–*Bam*HI sites modified from the pBY-JK1 construct (You and Shirako, 2010; **Figure 1A**). For construction of the cDNA clone of RNA2, named pBY-JT2, we performed RT-PCR using the TB166 primer for first-strand cDNA synthesis and TB163 and TB166 primers for PCR amplification. The purified 3.7-kb PCR product was digested with *Bam*HI and *Spe*I and then cloned into the *Bam*HI–*Spe*I sites of the pBY-JK2 construct (You and Shirako, 2010; **Figure 1A**). Both recombinant DNA procedures were carried out by standard methods using *Escherichia coli* strain MC1061, and the clones were verified by sequencing the plasmid inserts. All primers used for cloning are listed in Supplementary Table S1.

### Inoculation of Protoplasts and Leaves with *in vitro* Transcripts

RNA transcripts were synthesized *in vitro* from the linearized plasmids as described previously (Yamamiya and Shirako, 2000). Mesophyll protoplasts were prepared from 7-day-old barley seedlings according to procedures described previously (Ohsato et al., 2003; Li and Shirako, 2015). Approximately 5 × 10^5^ cells were transfected with capped *in vitro* transcripts of BaYMV RNA1 and RNA2 (4 μL transcripts of each) and subsequently incubated at 15°C for 66 h in the dark.

Inoculations of barley plants with *in vitro* transcripts were performed as described previously (Li and Shirako, 2015). Three to seven seedlings with two to three leaf stages were inoculated in each experiment. Inoculated plants were kept in a growth chamber at 15°C with 16 h of illumination.

### Construction of RNA1 Chimeras

**FIGURE 2:**
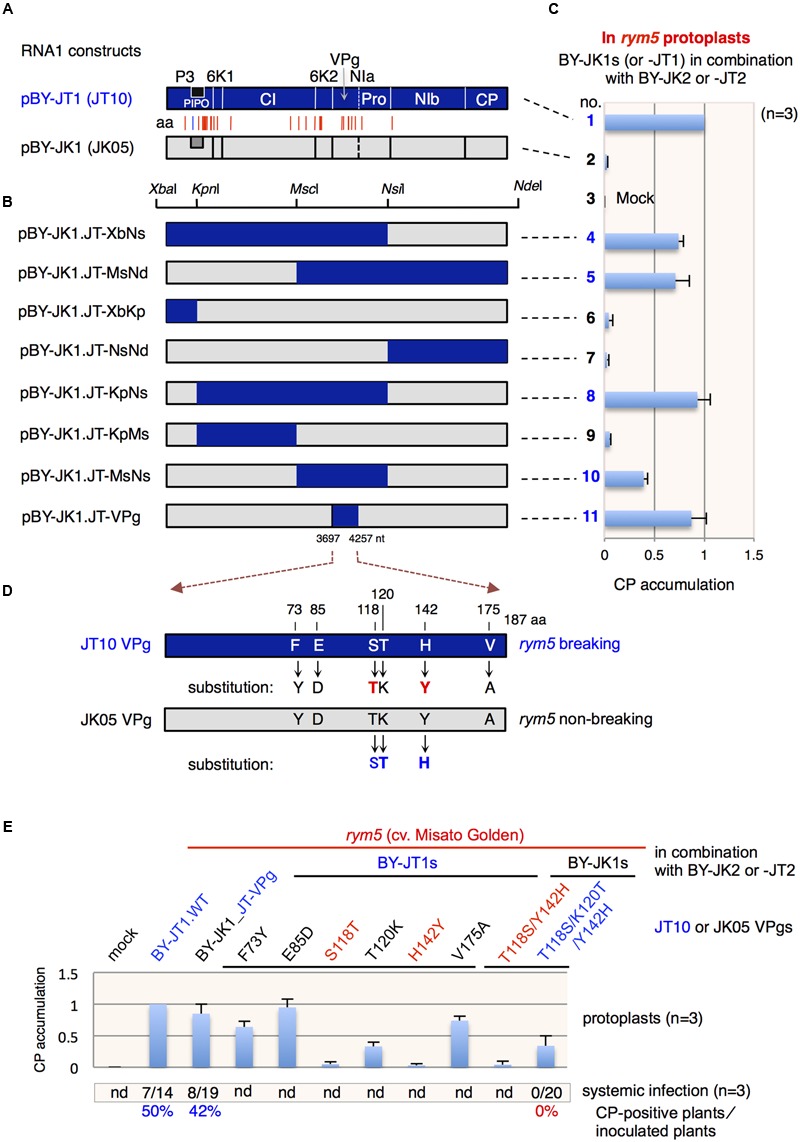
**Barley yellow mosaic virus (BaYMV) VPg is responsible for breakdown of *rym5* resistance in barley plants. (A)** Schematic representation of RNA1 cDNA clones, pBY-JT1 (JT10) and pBY-JK1 (JK05). The positions of amino acid (aa) differences between Tochigi isolate JT10 (*rym5*-breaking) and Kurashiki isolate JK05 (*rym5*-non-breaking) are indicated with vertical red lines. A vertical blue line indicates the position of one amino acid difference in the PIPO protein. (**(B)** Schematic representation of chimeric cDNA clones between pBY-JT1 (JT10) and pBY-JK1 (JK05). The unique restriction sites for chimera construction are shown. **(C)** BaYMV coat protein (CP) accumulation relative to the CP accumulation of the wild-type JT10 virus in *rym5* protoplasts transfected with chimeric RNA1 transcripts in addition to wild-type RNA2 transcripts (BY-JK2 or BY-JT2). **(D)** Schematic representation showing amino acid differences between JT10 VPg and JK05 VPg. The amino acid substitutions in each VPg are indicated with arrows. **(E)** Relative CP accumulation of JT10 or JK05 isolate having an amino acid mutation in VPg in *rym5* protoplasts. The total number of CP-positive plants in the upper leaves/the total number of inoculated plants and percentage of total plants showed viral systemic infection are presented below the graph. nd, not determined. CP accumulations in transfected protoplasts or upper systemic leaves of inoculated plants were detected by western blotting **(C,E)**. All values in bar graphs represent means with standard deviation (SD) from three independent experiments. Samples from *rym5* protoplasts inoculated with JT10 were used as the reference and the relative mean values of virus mutants were calibrated against those of references.

For the mapping analysis of viral determinants for overcoming *rym5* resistance, eight chimeric infectious cDNA clones were constructed for infectivity analysis: pBY-JK1.JT-XbNs [5′ untranslated region (UTR)–*Nsi*I region from pBY-JT1], pBY-JK1.JT-MsNd (*Msc*I–*Nde*I region from pBY-JT1), pBY-JK1.JT-XbKp (5′ UTR–*Kpn*I region from pBY-JT1), pBY-JK1.JT-NsNd (*Nsi*I–NdeI region from pBY-JT1), pBY-JK1.JT-KpNs (*Kpn*I–*Nsi*I region from pBY-JT1), pBY-JK1.JT-KpMs (*Kpn*I–*Msc*I region from pBY-JT1), pBY-JK1.JT-MsNs (*Msc*I–*Nsi*I region from pBY-JT1), and pBY-JK1.JT-VPg (VPg coding region from pBY-JT1; **Figure 2B**). These constructs were generated by replacements of the regions in Kurashiki isolate JK05 with corresponding regions of Tochigi isolate JT10. To generate pBY-JK1.JT-VPg, PCRs were performed in the presence of pBY-JK1 and pBY-JT1 as the templates. The resultant PCR products were purified, and they served as a mixed template to generate the fragment containing a *VPg* gene from Tochigi isolate JT10 in the background of Kurashiki isolate JK05. The final amplified fragments were purified and cloned into pBY-JK1 with *Afl*II and *Sal*I sites using an In-Fusion HD Cloning System CE Kit (Clontech Laboratories, USA). The primers used for this construction are listed in Supplementary Table S1. To generate the pBY-JK1.JT-MsNs construct, an *Msc*I–*Nsi*I fragment was released from pBY-JT1 and ligated into a similarly digested pBY-JK1. To generate the pBY-JK1.JT-KpNs construct, a *Kpn*I–*Nsi*I fragment was released from pBY-JT1 and ligated into a similarly digested pBY-JK1. To generate the pBY-JK1.JT-XbNs construct, an *Xba*I–*Nsi*I fragment was released from pBY-JT1 and ligated into a similarly digested pBY-JK1. To generate the pBY-JK1.JT-MsNd construct, an *Msc*I–*Nde*I fragment was released from pBY-JT1 and ligated into a similarly digested pBY-JK1. To generate the pBY-JK1.JT-KpMs construct, a *Kpn*I–*Msc*I fragment was released from pBY-JT1 and ligated into a similarly digested pBY-JK1. To generate the pBY-JK1.JT-XbKp construct, an *Xba*I–*Kpn*I fragment was released from pBY-JT1 and ligated into a similarly digested pBY-JK1. To generate the pBY-JK1.JT-NsNd construct, an *Nsi*I–*Nde*I fragment was released from pBY-JT1 and ligated into a similarly digested pBY-JK1.

pBY-JK1.JG-VPg, pBY-JK1.Y1A-VPg, and pBY-JK1.Y2A-VPg were constructed similarly to pBY-JK1.JT-VPg. All recombinant DNA procedures were carried out by standard methods using *E. coli* strain MC1061, and the clones were verified by sequencing the plasmid inserts.

### Construction of RNA2 Mutants

The GFP (green fluorescent protein)-expressing RNA2 mutant pBY-JT2.GFP (**Figure 1A**) was derived from pBY-JT2 in the same manner as pBY-JK2.GFP replacement of the P1 and P2 coding regions between the 5′- and 3′-UTRs of RNA2 with a *GFP* gene (You and Shirako, 2010). The primers used for construction are listed in Supplementary Table S1.

Two eIF4E-expressing RNA2 mutants, pBY-JK2.P12/KoA_eIF4E(NIb/CP) and pBY-JK2.P12/KoA_eIF4E(P1/P2), were constructed and used in this study (**Figure 4A**). PCRs were performed in the presence of pBY-JK2 and the KoA *eIF4E* gene as separate templates (Li and Shirako, 2015). The resultant PCR products were purified, and they served as a mixed template for generation of the final expected fragments. The fragments were subsequently digested and ligated into pBY-JK2. All recombinant DNA procedures were carried out by standard methods using *E. coli* strain MC1061, and the clones were verified by sequencing the plasmid inserts. The primers used for each construct are listed in Supplementary Table S1.

### P2 and eIF4E Antiserum Production

BaYMV P2 and barley eIF4E proteins were prepared by fusing proteins with glutathione *S*-transferase (GST) in *E. coli* cells (strain MC1061), as described by You and Shirako (2010). One rabbit was immunized for 2 mg of each purified recombinant fusion protein (GST:P2 and GST:eIF4E).

### Western Blot Analysis

**FIGURE 3:**
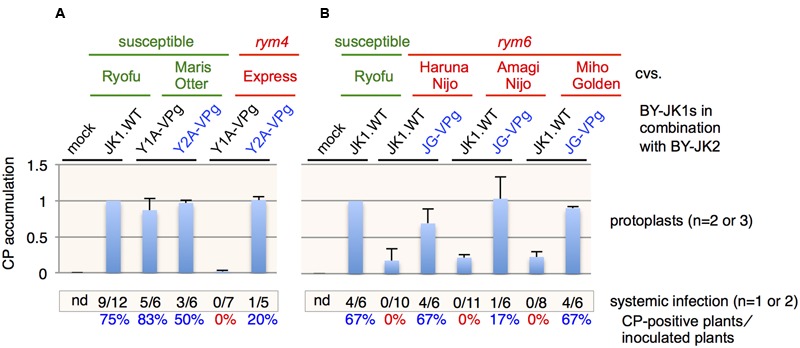
**Barley yellow mosaic virus (BaYMV) VPg is responsible for breakdown of *rym4* and *rym6* resistances. (A,B)** Relative BaYMV coat protein (CP) accumulation of the JK05 isolate with VPg replacement in *rym4* **(A)** and *rym6* **(B)**. All values in bar graphs represent means with SD from two or three independent experiments. Samples from Ryofu protoplasts inoculated with JK05 were used as the reference.

Western blot analysis was performed as described previously (Yamaguchi and Shirako, 2002). Polyclonal antibodies against BaYMV CP (coat protein), P2, and barley eIF4E were used as the primary antibodies. Each virus inoculation test on protoplasts or whole plants was at least triplicated, except for inoculation of BY-JK1.Y1A-VPg and Y2A-VPg mutants, which were duplicated in *rym4* protoplasts, and inoculation of BY-JK1, BY-JK1.Y1A-VPg, Y2A-VPg, and JG-VPg mutants, which were only performed once or twice in *rym4* and *rym6* plants (**Figure 3**). Positive (BaYMV-infected susceptible plants) and negative (the mock plants) control samples were always included in each blot.

To quantify the levels of CP accumulation in the protoplasts, the CP bands on all of the blots were scanned using ImageJ 1.42q software (Wayne Rasband National Institutes of Health, USA). The intensity of CP band of each sample was normalized with the intensity of the rubisco large subunit band of the corresponding sample, and the relative mean scores were calibrated against those of positive controls as described previously (You and Shirako, 2013).

### Barley eIF4E Protein Homology Modeling

The amino acid sequence of barley KoA eIF4E was submitted to the homology-modeling server SWISS-MODEL (Arnold et al., 2006; Biasini et al., 2014)^1^. Construction of the three-dimensional structure of this protein was based on sequence homology to wheat eIF4E (PDB code: 2idr). The predicted structure was visualized with the software Deep View/Swiss-PdbViewer v4.1.0 (Johansson et al., 2012).

## Results

### Construction of an Infectious cDNA Clone from a *rym5*-Breaking BaYMV Isolate

The full-length nucleotide sequences of RNA1 and RNA2 of BaYMV JT10 (*rym5*-breaking isolate) were determined and deposited in the GenBank database with accession numbers AB920780 for RNA1 (7642 nt) and AB920781 for RNA2 (3585 nt). The full-length cDNA clones of JT10 were constructed and named pBY-JT1 and pBY-JT2 (**Figure 1A**) for RNA1 and RNA2, respectively. The replicative ability of JT10 RNA transcripts (BY-JT1 RNA1 and BY-JT2 RNA2) from the two clones was examined in protoplasts isolated from *rym5*-carrying barley cultivars (Mikamo Golden and Misato Golden). The protoplasts isolated from barley cultivar Ryofu were used as a susceptible control. The protoplasts were transfected with JT10 transcripts and then incubated at 15°C for 66 h in the dark. After incubation, the protoplasts were harvested and subjected to western blot analyses using a CP antiserum (You and Shirako, 2010). CP accumulations were detected in all the tested protoplasts (**Figure 1B**), indicating virus multiplication. To monitor visually the virus infection in protoplasts, the P1–P2 polyprotein coding region in pBY-JT2 was replaced with a *GFP* gene-coding sequence (pBY-JT2.GFP; **Figure 1A**). GFP fluorescence was observed in both susceptible and *rym5* protoplasts transfected with BY-JT1 RNA1 + BY-JT2.GFP RNA2, but not in *rym5* protoplasts transfected with BY-JK1 RNA1 + BY-JK2.GFP RNA2 (**Figure 1C**), confirming the infectious nature of the transcripts.

Next, the systemic infectivity of JT10 RNA1 and RNA2 was examined at the whole plant level through manual inoculation. Yellow mosaic leaf symptoms were observed in susceptible (cultivar Ryofu) and *rym5* (cultivars Mikamo Golden and Misato Golden) plants at 5–7 weeks post-inoculation (w.p.i.; **Figure 1D** and data not shown). RT-PCR/sequencing analysis of virus progenies confirmed that the JT10 transcripts were systemically infectious to the *rym5* cultivars (data not shown). For comparison, the cultivar Ryofu plants inoculated with JK05 RNA transcripts (BY-JK1 RNA1 and BY-JK2 RNA2 of a *rym5*-non-breaking BaYMV isolate; You and Shirako, 2013) developed yellow mosaic symptoms 3–5 weeks faster than those inoculated with the JT10 virus (data not shown).

### VPg is the Determinant Viral Protein for Breaking *rym5* Resistance

Previous research showed that BaYMV RNA1, which encodes a set of proteins including the RNA polymerase, could autonomously replicate in barley protoplasts while RNA2 needs RNA1 for replication (You and Shirako, 2010; Li and Shirako, 2015). Thus, we speculated that BaYMV RNA1 encoded the most important factor(s) responsible for breaking eIF4E-mediated (*rym5*) resistance. Additionally, as replacing the coding region in RNA2 with the *GFP* gene (**Figure 1A**) does not affect JT10 ability to multiply in *rym5* protoplasts (**Figure 1C**), the viral factor(s) responsible for breaking *rym5* resistance thus likely reside in BaYMV RNA1. Sequence comparison showed that within RNA1, the JT10 isolate has 29 amino acid differences with the *rym5*-non-breaking JK05 isolate (You and Shirako, 2013). Notably, the amino acid substitutions were present in almost all of the coding proteins except for CP (**Figure 2A**).

To identify the viral factor(s) involved in breaking *rym5* resistance, partial DNA fragments derived from pBY-JT1 were used to replace the corresponding sequences in a cDNA clone derived from JK05 RNA1 that was generated previously (You and Shirako, 2010; here renamed as pBY-JK1 and pBY-JK2 for RNA1- and RNA2-derived clones, respectively). Seven chimeras were constructed by utilizing the available unique restriction enzyme sites (**Figure 2B**). Leaf protoplasts isolated from cultivar Misato Golden (*rym5*) plants were transfected with the mixtures of *in vitro* transcripts synthesized using pBY-JK1-based chimeras and pBY-JK2. Western blotting indicated that four chimeric viruses (BY-JK1.JT-XbNs, BY-JK1.JT-MsNd, BY-JK1.JT-KpNs, and BY-JK1.JT-MsNs) were able to accumulate in *rym5* protoplasts, although the level of the BY-JK1.JT-MsNs was lower than that of other mutants, probably due to the inhibiting effect of the chimeric sequence on virus accumulation (**Figure 2C**). Those infectious transcripts similarly contained the central region of the JT10 genome, which encodes for 6K2 (6 kDa protein 2), VPg and NIa (nuclear inclusion a proteinase; **Figure 2B**). When only the VPg portion of the JT10 genome was used to replace the corresponding region in pBY-JK1, the chimeric transcripts (BY-JK1.JT-VPg) were infectious in *rym5* protoplasts (**Figure 2C**) and also in whole plants, in which a mild mosaic symptom appeared and CP accumulations were detected in upper systemic leaves at 4 w.p.i. (**Figure 2E**; data not shown). These observations confirm that VPg is responsible for breaking eIF4E-mediated resistance in barley plants.

### Ser-118, Thr-120, and His-142 of VPg are Critical for Overcoming *rym5* Resistance

Sequence alignment showed that six amino acid residues at positions 73, 85, 118, 120, 142, and 175 in JT10 VPg differ from the corresponding residues in JK05 VPg (**Figure 2D**; **Table 1**). To identify precisely which key amino acid(s) of VPg are important for breaking *rym5* resistance, each of those six amino acids in the pBY-JT1 background was substituted into the same residues as encoded in the JK05 VPg, and then the infectivity of each virus mutant (with the presence of JT1 RNA2) was examined in *rym5* protoplasts and whole plants (cultivar Misato Golden). Western blotting showed that substitution of Ser-118 and His-142 into Thr (S118T) and Tyr (H142Y), respectively, abolished JT10 ability to infect *rym5* protoplast, while substitution of Thr-120 into Lys (T120K) largely reduced JT10 accumulation in *rym5* protoplasts (**Figure 2E**). In contrast, mutation of Phe-73, Glu-85, and Val-175 (F73Y, E85D, and V175A, respectively) had no or partial effects on JT10 accumulation in *rym5* protoplasts (**Figure 2E**). These results indicate that Ser-118 and His-142 as well as Thr-120, to a lesser degree, are critical for overcoming *rym5* resistance, while Phe-73, Glu-85, and Val-175 are less important for breaking *rym5* resistance at the intracellular level. Our preliminary results showed that the JT10 mutants with the substitution of Phe-73 or Glu-85 did not cause any systemic symptom in *rym5* plants (data not shown). This may suggest that these residues are critical for viral systemic infection throughout whole plants.

**Table 1:** Amino acid differences of VPg proteins from barley yellow mosaic virus (BaYMV) JK05, JT10, JG10, Y1A, and Y2A isolates.

BaYMV isolates^a^	Amino acid position in VPg																				
	42	73	85	88	113	114	118	120	122	132	134	142	151	165	167	169	174	175	179	181	183
JK05	S	Y	D	K	Q	M	T	K	I	H	D	Y	I	S	K	T	G	A	T	T	D
JT10		**F**^b^	**E**				**S**	**T**				**H**						**V**			
JG10												H		P	Q	A		V			
Y1A	A			R	K	L		N	V	K	E	F	V	V	V	G	E		A	A	E
Y2A				R	K	L		N		N	E	F	V	V	R	G	E		A	A	E

*^a^JK05, rym4/5/6-non-breaking isolate (You and Shirako, 2013); JT10, rym5-breaking isolate (this study); JG10, rym6-breaking isolate (this study); Y1A, rym4-non-breaking isolate (Kühne et al., 2003); Y2A, rym4-breaking isolate (Kühne et al., 2003).*

*^b^The characters in bold indicate the amino acids in JT10 VPg that are different from those of JK05 VPg.*

In a reciprocal experiment, JK05 was able to infect *rym5* protoplasts only when Thr-118, Lys-120, and Tyr-142 were simultaneously substituted (triple mutation, T118S/K120T/Y142H) but not when only Thr-118 and Tyr-142 were simultaneously substituted (double mutation, T118S/Y142H; **Figure 2E**). The accumulation level of JK05 with the triple mutations was low in *rym5* protoplasts (**Figure 2E**), which is consistent with the previous observation (using JT10). Moreover, JK05 with the triple mutations was unable to infect the whole plants (**Figure 2E**), in line with the notion that the two other amino acid residues at the position of 73 and 85 in VPg might be essential for viral systemic infection, although they are partially important for breaking the resistance at the intracellular level.

### VPg is Also the Determinant Protein Responsible for Breaking *rym4* and *rym6* Resistances

Since *rym4*/*5*/*6* are allelic with similar *eIF4E* genes (Kanyuka et al., 2005; Stein et al., 2005), it is possible that VPg is also the determinant protein for breaking *rym4* and *rym6* resistances. In Europe, *rym4* resistance has been used widely for barley breeding in recent decades (Kühne, 2009). BaYMV isolates from German Y2A but not Y1A were able to infect *rym4* plants in the field (Kühne et al., 2003). When *VPg* genes derived from Y2A and Y1A isolates were used to replace the *VPg* gene in pBY-JK1, JK05 Y2A-VPg but not JK05 Y1A-VPg could infect *rym4* protoplasts and plants (cultivar Express), whereas both chimeric viruses could infect susceptible barley plants (cultivar Maris Otter; **Figure 3A**). The BaYMV JK05 isolate could accumulate to low levels in *rym6* protoplasts (cultivars Haruna Nijo, Amagi Nijo, or Miho Golden) but could not systemically infect *rym6* plants (You and Shirako, 2013; **Figure 3B**). The BaYMV JG10 isolate is a *rym6*-breaking isolate from *rym6* plants (cultivar Haruna Nijo) in Gumma, Japan in 2010 (see **Table 1** for its VPg amino acid sequence; Shirako and Li, unpublished data). JK05 JG10-VPg accumulated to high levels in *rym6* protoplasts and systemically infected all three varieties carrying *rym6* (**Figure 3B**). Taken together, BaYMV VPg is also the determinant protein for breaking *rym4* and *rym6* resistances.

### Expression of *eIF4E* gene Derived from a Susceptible Barley Cultivar Enables JK05 Multiplication in *rym5* Protoplasts

**FIGURE 4:**
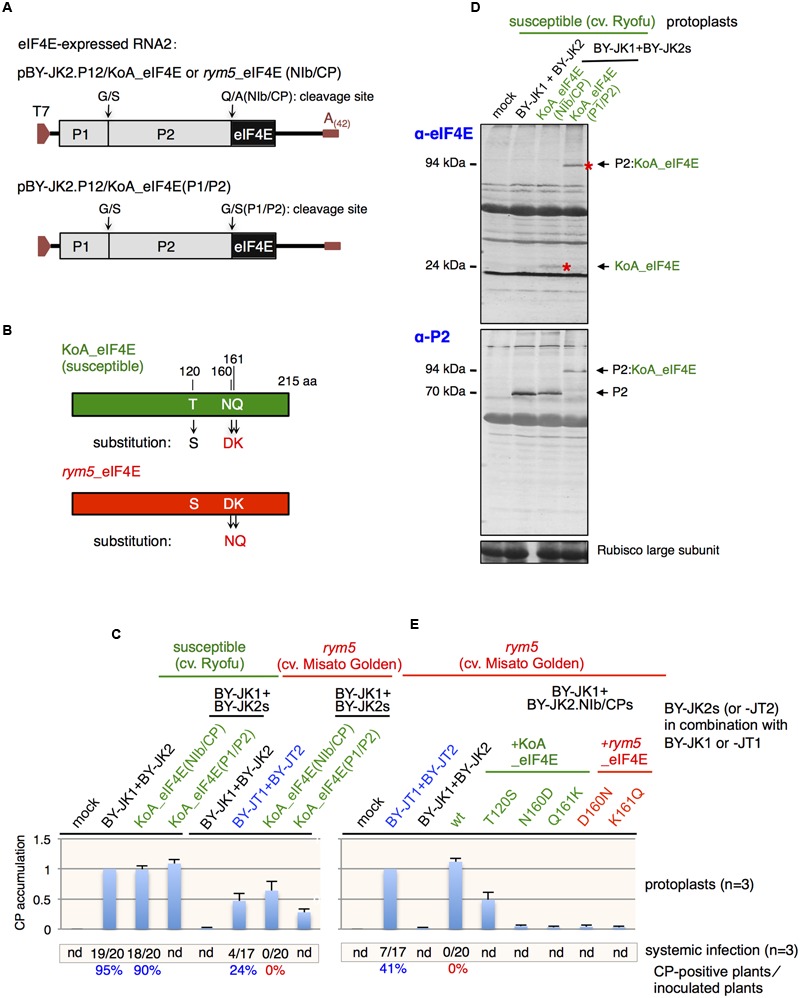
**Expression of eIF4E from viral genome. (A)** Schematic representation of eIF4E-expressing JK05 RNA2 constructs. **(B)** Schematic representation showing major amino acid differences between KoA (susceptible) and *rym5* (resistance) eIF4E alleles. The amino acid substitutions in each eIF4E are indicated with arrows. **(C,E)** Barley yellow mosaic virus (BaYMV) coat protein (CP) accumulation in susceptible and *rym5* protoplasts transfected with eIF4E-expressing JK05 RNA2 (BY-JK2) transcripts in addition to JK05 RNA1 (BY-JK1) transcripts. All values in bar graphs represent means with SD from three independent experiments. Samples from Ryofu or *rym5* protoplasts inoculated with JK05 **(C)** or JT10 **(E)** were used as the reference. **(D)** Western blot analysis of barley eIF4E and BaYMV P2 accumulation in protoplasts from cultivar Ryofu (susceptible) transfected with eIF4E-expressing JK05 RNA2 (BY-JK2) transcripts plus JK05 RNA1 (BY-JK1) transcripts. The red asterisks mark the bands of eIF4E and P2:eIF4E fusions on the blots.

Considering that the compatibility between VPg and host eIF4E plays an important role in determining BaYMV ability to infect barley plants, we anticipated that the expression of *eIF4E* gene derived from a susceptible barley cultivar could facilitate the accumulation of non-breaking isolates in *rym5* protoplasts. To this end, the *eIF4E* gene derived from susceptible cultivar KoA (KoA eIF4E) was inserted into JK05 RNA2 (pBY-JK2) downstream of the *P2* gene with the addition of either the NIb/CP or the P1/P2 protease cleavage site to release eIF4E from the polyprotein or to express the P2 fusion product (P1 proteinase most probably only acts in *cis*; **Figures 4A,B**). Protoplasts from susceptible (cultivar Ryofu) and *rym5* (cultivar Misato Golden) plants were transfected with JK05 RNA1 and eIF4E-expressing RNA2 transcripts. As expected, CP accumulations were detected in *rym5* protoplasts after transfection with either JK05 RNA1 + JK05 RNA2 KoA_eIF4E(NIb/CP) or + JK05 RNA2 KoA_eIF4E(P1/P2) transcripts (**Figure 4C**). The CP accumulations of those KoA_eIF4E-expressing viruses in *rym5* protoplasts were not as abundant as those in susceptible protoplasts but were rather comparable to JT10 accumulations in *rym5* protoplasts (**Figure 4C**). These results demonstrated that expression of KoA eIF4E from viral genome, to some extent, could assist incompatible JK05 (*rym5*-non-breaking isolate) in accumulating in *rym5* protoplasts. Note that although both KoA eIF4E-carrying transcripts were infectious in *rym5* protoplasts, as expected, free eIF4E (∼24 kDa) was efficiently released by the NIb/CP but not the P1/P2 cleavage sites (**Figure 4D**). Thus, eIF4E also could be functional in the form of a P2-eIF4E fusion protein (∼94 kDa). Nevertheless, expression of KoA eIF4E from the viral genome did not enable JK05 infection in whole plants (**Figure 4C**), indicating that some other resistance factor(s) should be involved in *rym5* plants.

Sequence alignment showed that the three amino acid residues at positions 120 (Thr), 160 (Asn), and 161 (Gln) in eIF4E from *rym5*-carrying cultivars are different from those corresponding residues in eIF4E from susceptible cultivars (**Figure 4E**; **Table 2**). Substitution of both Asn-160 and Gln-161 into Asp and Lys (N160D and Q161K), respectively, abolished KoA eIF4E activity to facilitate JK05 infection in *rym5* (cultivar Misato Golden) protoplasts, while substitution of Thr-120 into Ser (T120S) only reduced virus accumulation (**Figure 4E**). Nevertheless, substitutions of either Asp-160 to Asn (D160N) or Lys-161 to Gln (K161Q) in *rym5* eIF4E were not sufficient to enable JK05 accumulation in *rym5* protoplasts (**Figure 4E**). Thus, the simultaneous presence of both Asn-160 and Gln-161 in *rym5* eIF4E might be required for facilitating BaYMV infection.

**Table 2:** Amino acid differences of eIF4E proteins from several barley varieties carrying different resistance alleles.

Varieties	Resistance alleles	Amino acid position in eIF4E^a^									
		53	57	118	120	160	161	198	205	206	208
Ryofu	–	P	S	K	T	N	Q	V	S	D	G
KoA	–							D			S
Express	*rym4*		F	T					F	G	A
Mikamo Golden	*rym5*				**S**^b^	**D**	**K**				
Misato Golden	*rym5*				**S**	**D**	**K**				
Amagi Nijo	*rym6*	S				H					
Miho Golden	*rym6*	S				H					
Haruna Nijo	*rym6*	S				H					

*^a^The sequence information of barley eIF4E was referred to by Kanyuka et al. (2005), by Stein et al. (2005), and Li and Shirako (2015) and sequenced again in this study (GenBank accession numbers: Ryofu: AB592971; Mikamo Golden: AB592982; Haruna Nijo: AB592977).*

*^b^The characters in bold indicate the amino acids in rym5 eIF4E that are different from those of eIF4E proteins from the other barley varieties.*

## Discussion

In the present study, viral factor(s) responsible for breaking *rym5* resistance were identified by genomic mapping analysis using infectious cDNA clones derived from BaYMV JK05 (*rym5*-non-breaking) and JT10 (*rym5*-breaking) isolates (**Figures 1** and **2**). Along with the presence of JK05 RNA2, the JK05 RNA1 chimera (BY-JK1.JT-VPg) with the JK05 *VPg* gene replaced with that of JT10 isolate accumulated in *rym5* protoplasts and was infectious to *rym5* plants at the whole plant level (**Figure 2**). Likewise, the RNA1 VPg chimeras with the JK05 *VPg* gene replaced with those of *rym4* and *rym6* resistance-breaking isolates (Y2A and JG) each individually disrupted the corresponding resistance at the whole plant level (**Figure 3**; and summarized in **Table 3**). Thus, BaYMV VPg is the determinant protein responsible for breaking eIF4E-mediated *rym4*/*5*/*6* resistance in barley plants. Furthermore, we tried to identify precisely the key amino acid(s) of VPg required for breaking these eIF4E-mediated resistances. Substitutions of at least three amino acids at the positions of 118, 120, and 142 in VPg of *rym5*-non-breaking isolate JK05 are required for overcoming *rym5* resistance in protoplasts, but they were not sufficient for the establishment of viral systemic infection in *rym5* plants (**Figure 2**). These results indicate that mutations of numerous amino acid residues in VPg are required for complete breakdown of eIF4E-mediated resistance in barley plants. VPg has some typical features of intrinsically disordered proteins and is known to be linked to the genome of some positive-sense RNA plant viruses (e.g., the members of *Potyviridae* and *Secoviridae* families and *Sobemovirus, Polerovirus*, and *Enamovirus* genera) and vertebrate ones (e.g., the members of *Picornaviridae* and *Caliciviridae* families; Jiang and Laliberté, 2011). In potyviruses, VPg is a hub protein that interacts with several proteins both of viral and host origin, likely including eIF4E (see below), and appears to be involved in diverse viral processes, such as translation, replication, cell-to-cell and/or long-distance movement (Jiang and Laliberté, 2011; Revers and García, 2015).

**Table 3:** Summary of accumulation and systemic infectivity of barley yellow mosaic virus (BaYMV) VPg mutants in *rym4*/*5*/*6* protoplasts or plants.

Inocula (BaYMV)		Protoplasts/plants^a^		
RNA1	RNA2	*rym4*	*rym5*	*rym6*
BY-JK1	BY-JK2	+/+	-/-	+/-
BY-JT1	BY-JT2	+/+	+/+	+/+
BY-JK1.JT-VPg	BY-JK2	+/+	+/+	+/+
BY-JK1.JG-VPg	BY-JK2	NT/+	-/-	+/+
BY-JK1.Y1A-VPg	BY-JK2	-/-	-/-	+/NT
BY-JK1.Y2A-VPg	BY-JK2	+/+	-/-	+/NT

*^a^+, coat protein (CP) could be detected from the protoplasts or plants; -, no CP was detected from the protoplasts or plants; NT, not tested.*

The role of barley eIF4E during BaYMV infection was examined by the expression of host eIF4E from the viral genome RNA. When an eIF4E derived from a susceptible cultivar was expressed from JK05 RNA2, CP accumulation was detected by western blot analysis (**Figure 4**), demonstrating that the presence of compatible eIF4E enables the multiplication of a *rym5*-non-breaking BaYMV isolate in *rym5* cells. In the case of potyviruses, the host eIF4E has been shown to affect virus infection, but there is no direct evidence that shows eIF4E function in virus multiplication at the intracellular level (Schaad et al., 2000; Gao et al., 2004). Our study provided the experimental evidence showing host eIF4E functions in plant virus multiplication at the intracellular level, although it remains unclear how eIF4E participates in this process. The current models for the eIF4E roles in potyvirus infection at the intracellular level proposes that eIF4E acts in concert with VPg and other viral (such as P1 and HC-Pro) and host [such as eIF4G and poly(A)-binding protein] factors to facilitate viral genome replication, translation and/or safeguarding virus translation/replication (Wang and Krishnaswamy, 2012; Mäkinen and Hafrén, 2014). In addition, eIF4E is also proposed to be involved in potyviral intracellular trafficking, cell-to-cell (or long-distance) movement via interaction with VPg, CI protein, and eIF4G (Wang and Krishnaswamy, 2012; Mäkinen and Hafrén, 2014). Interestingly, our results provide the notion that barley eIF4E also plays a role(s) in BaYMV cell-to-cell movement and/or systemic virus accumulation because the substitutions of multiplication-related amino acids in VPg from the non-breaking isolate were insufficient for breaking barley eIF4E-mediated resistance at the whole plant level (**Figure 2**). As progression of BaYMV infection is rather slow in barley plants, it is difficult to observe cell-to-cell movement processes, such as those observed for pea seed-borne mosaic virus (PSbMV, a potyvirus) in pea plants (Gao et al., 2004).

**FIGURE 5:**
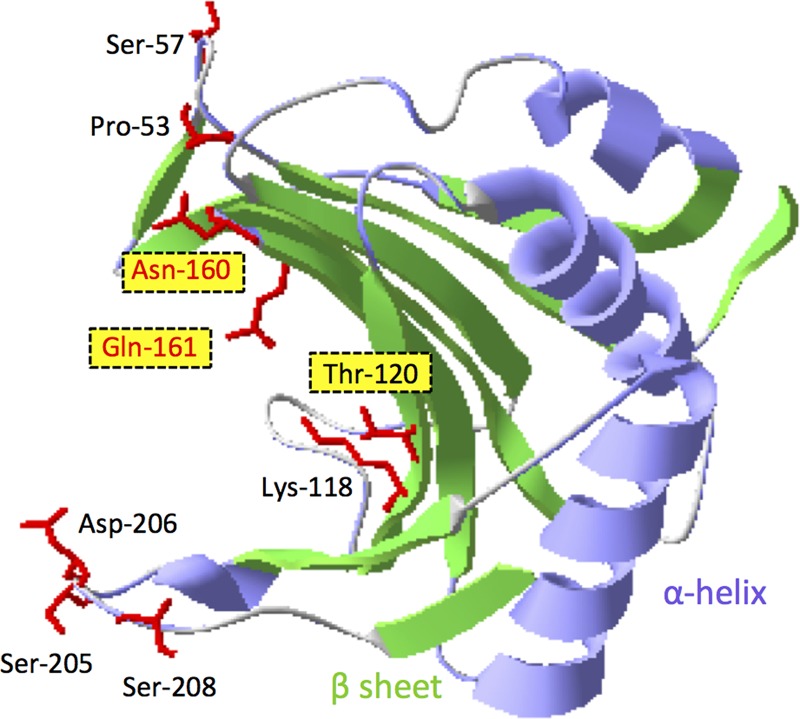
**Three-dimensional (3D) model of barley KoA eIF4E protein.** The putative 3D structure of KoA eIF4E was predicted by the homology-modeling server SWISS-MODEL. The N-terminal 39 amino acids of KoA eIF4E are invisible in this model. The putative cap-binding “pocket” is located at the left side of the eIF4E molecule. The amino acid residues of KoA eIF4E highlighted with the red side chains indicate the positions of amino acid differences among the eIF4E proteins encoded by *rym4, rym5*, and *rym6* plants (see **Table 2**). The residues highlighted with yellow boxes were mutated in this study. The 3D image was generated using Swiss-PdbViewer.

Our study indicates that the compatibility between VPg and eIF4E proteins is required for virus multiplication (and virus cell-to-cell movement), suggesting that between barley eIF4E and BaYMV VPg there might be a direct interaction or an indirect interaction with an interposed viral or host factor(s). However, a direct or indirect interaction between these two proteins was not detected by a yeast two-hybrid (Y2H) system or by co-immunoprecipitation assays (Li and Shirako, unpublished data). The inability to detect a VPg–eIF4E interaction might be due to a likely occurred misfolding of VPg to interact with eIF4E in Y2H-system, the low temperature requirement for BaYMV replication (You and Shirako, 2012), the instability of host eIF4E (Monzingo et al., 2007) or a possible occurrence of a weak interaction below detection limit. The failure to demonstrate the interaction between pea eIF4E and VPg encoded by PSbMV, which is also a low temperature-adapted virus (Congdon et al., 2016; Jones, 2016), was reported previously (Gao et al., 2004). Nonetheless, we speculate that interaction between BaYMV VPg and eIF4E does exist in host barley. The amino acid substitutions in barley eIF4E may disrupt its interaction with VPg and then result in resistance to BaYMV infection. Compared to an eIF4E from a susceptible cultivar, five substitutions at amino acid positions 57, 118, 205, 206, and 208 were found in *rym4* eIF4E; three substitutions at positions 120, 160, and 161 were found in *rym5* eIF4E; and two substitutions at positions 53 and 161 were found in *rym6* eIF4E (Kanyuka et al., 2005; Hofinger et al., 2011; **Table 2**; Li and Shirako, unpublished data). According to the three-dimensional model of barley eIF4E (Kanyuka et al., 2005; Stein et al., 2005; **Figure 5**), all amino acid substitutions are located at or near the cap structure binding domain. As predicted in wheat eIF4E (Monzingo et al., 2007), amino acids at positions 160 and 161 are close to two key amino acids, 158 and 163, which are predicted to be located at a cap structure binding region, suggesting that amino acids at position 160 and 161 also may be involved in the binding of the RNA cap structure. Substitutions in either of these two positions may result in resistance to viral infection, as occurred in *rym5* and *rym6* (**Table 2**). In addition, the amino acid at position 118 was important for stabilizing the structure of the protein, and the substitution at this position, as occurred in *rym4* eIF4E, also may affect the function of eIF4E (**Table 2**).

The infectivity assays carried out using both protoplasts and whole plants were useful for elucidating resistance mechanisms. It becomes clear whether the step in the viral multiplication or movement was blocked during the resistance responses. In many examples of potyvirus infection, compatibility between VPg and eIF4E has been shown. Our present data reveal that both barley eIF4E and BaYMV VPg function in virus multiplication and/or, presumably, virus cell-to-cell movement. The knowledge regarding amino acid residues in eIF4E and VPg that are critical for virus infection can be utilized in the crop breeding programs as well as in the surveillance of emerging resistance-breaking virus variants. Lastly, this work provides a basis for further understanding of other recessive resistance mechanisms in plants.

## Author Contributions

HL and YS conceived the work plan, conducted the experiments and data analysis, and drafted the first draft of the manuscript. TK and YS contributed materials and reagents. HL and HK drew tables and figures. First draft of the manuscript was edited and approved by all authors.

## Conflict of Interest Statement

The authors declare that the research was conducted in the absence of any commercial or financial relationships that could be construed as a potential conflict of interest.

## Footnotes

1 https://swissmodel.expasy.org/
