# Supplementary material for: Barley Yellow Mosaic Virus VPg Is the Determinant Protein for Breaking eIF4E-Mediated Recessive Resistance in Barley Plants
Source: Front Plant Sci. 2016 Sep 30;7:1449. doi: 10.3389/fpls.2016.01449 (PMC5043020; doi:10.3389/fpls.2016.01449)
Supplement: Supplementary file 1 [file Table_1.DOCX]

Supplementary Material

Barley yellow mosaic virus VPg is the determinant protein for breaking eIF4E-mediated recessive resistance in barley plants

**Huangai Li ^＊^, Hideki Kondo, Thomas Kühne, Yukio Shirako^＊^**

*** Correspondence:**Huangai Li
huangaili@yahoo.com

Yukio Shirako
ushirako@mail.ecc.u-tokyo.ac.jp

# Supplementary Table

**Supplementary Table 1.** Primers used for this study^a^.

| Constructs | ID | Sequence (5'→3') |
| --- | --- | --- |
| pBY-JT1 | TB87 | ctc/TCTAGA/**TAATACGACTCACTATA**/G/AAAATAAAACAACCCTAAAC |
|  | TB162 | aga/GGATCC/(T)_42_^c^/ATTACCTTCTGGTACTC |
| pBY-JT2 | TB163 | aga/GGATCC/**TAATACGACTCACTATA**/G/AAAATAAAACAACCCTACACCA |
|  | TB166 | cac/ACTAGT/(T)_42_/GTCACATTTCCTGTGTAC |
| pBY-JT2.GFP | TB163 | aga/GGATCC/**TAATACGACTCACTATA**/G/AAAATAAAACAACCCTACACCA |
|  | TB170 | ***TTTGCTAGCCAT***/GATGGAGGGTTTGAAAGC |
|  | TB97 | CCCTCCATC/***ATGGCTAGCAAAGGAGA*** |
|  | TB169 | ATGAGAAAT/***TCAGTTGTACAGTTCAT*** |
|  | TB168 | ***TACAACTGA***/ATTTCTCATCACAGCAG |
|  | TB166 | cac/ACTAGT/(T)_42_/GTCACATTTCCTGTGTAC |
| pBY-JK1.JT-VPg | TB146 | GCAAGTGGCGCGAGCATC |
|  | TB204 | GTTTCCCTTACCTTCAAAGACCAATTTTGTC |
|  | TB203 | ATTGGTCTTGAAGGTAAGGGAAACAAGTAC |
|  | TB147 | CCAACTCGGGTTGCGACG |
| pBY-JT1.VPg_F73Y | TB207 | TCAGTGGTGtAGAAAATAGCTTCCAGAA |
|  | TB206 | CTATTTTCTaCACCACTGAAGGCGATGA |
| pBY-JT1.VPg_E85D | TB209 | CTTATTGGGaTCTGCTGTTCGGAAAAAC |
|  | TB208 | AACAGCAGAtCCCAATAAGGACATGAAC |
| pBY-JT1.VPg_S118T | TB211 | CCGTGGCTGtTTCTTCTAGCATCTGCCG |
|  | TB210 | CTAGAAGAAaCAGCCACGGTTATTATCA |
| pBY-JT1.VPg_T120K | TB151 | TTTGATAATAACCtTGGCTGTTTCTTC |
|  | TB150 | GAAGAATCAGCCAaGGTTATTATCAAA |
| pBY-JT1.VPg_H142Y | TB213 | GCTTTAGATaGTCTGGATCATGCTGTGA |
|  | TB212 | GATCCAGACtATCTAAAGCAGAATGGGT |
| pBY-JT1.VPg_V175A | TB215 | CCAAACTCAgCTCCAAGATCGTAATCCG |
|  | TB214 | ATCTTGGAGcTGAGTTTGGCACCGACAC |
| pBY-JK1.VPg_T118S /H142Y | TB133 | AATAACCTTGGCTGaTTCTTCTAGCAT |
|  | TB132 | ATGCTAGAAGAAtCAGCCAAGGTTATT |
|  | TB137 | ATTCTGCTTTAGATgGTCTGGATCATG |
|  | TB136 | CATGATCCAGACcATCTAAAGCAGAAT |
| pBY-JK1.VPg_T118S/K120T/Y142H | TB202 | ATAATAACCgTGGCTGaTTCTTCTAGCATCTG |
|  | TB201 | CTAGAAGAAtCAGCCAcGGTTATTATCAAAGA |
| pBY-JK1.Y1A-VPg | TB41 | GAACATACAAGCGAGCA |
|  | TB187 | GTTTCCCTTGCCTTCAAGACCAATTTTGTC |
|  | TB185 | ATTGGTCTTGAAGGCAAGGGAAACAAATAC |
|  | TB190 | AACTCGGGTTGCGACGTCGACCCCAACTTGTGACAATAATATCCCAGTAGAGGCCCCAAAGCTTATTTCATC |
| pBY-JK1.Y2A-VPg | TB41 ^b^ |  |
|  | TB187 ^b^ |  |
|  | TB185 ^b^ |  |
|  | TB186 | AACTCGGGTTGCGACGTCGACCCCAACTTGTGACAATAATATCCCAGTAGAGGCCTCAAGCGTTATTTCATC |
| pBY-JK1.JG-VPg | TB204 ^b^ |  |
|  | TB203 ^b^ |  |
|  | TB205 | AACTCGGGTTGCGACGTCGACCCCAACTTGTGACAATAATATCCCAGTAGAGGCTTCGAGCGTGATGTCATC |
| pBY-JK1.Y1A-VPg_K132N | TB222 | AATTTCCAT/gTT/GTGCGCAGTTCCCTTC |
|  | TB221 | TGCGCAC/AAc/ATGGAAATTTCACAGCAT |
| pBY-JK1.Y2A-VPg_N132K | TB224 | AATTTCCAT/cTT/GTGCGCAGTTCCCTTC |
|  | TB223 | TGCGCAC/AAg/ ATGGAAATTTCGCAGCAT |
| pBY-JK1.VPg_Y142H | TB137 ^b^ |  |
|  | TB136 ^b^ |  |
| pBY-JK1.JG-VPg_H142Y | TB213 ^b^ |  |
|  | TB212 ^b^ |  |
| pBY-JK2.P12/KoA_eIF4E(NIb/CP) | TB65 | CGAACTTCAGCTTGAAG |
|  | TB270 | ***AGCTTGCAGCCAAATTTCATC***/GACTCGAGGGCGCCGGAAGAG |
|  | TB269 | ***GATGAAATTTGGCTGCAAGCT***/ATGGCGGAGGACACGGAGACG |
|  | TB129 | GTTCGTCAGACTACAAC |
| pBY-JK2.P12/KoA_eIF4E(P1/P2) | TB182 | CGCCAT/***TGAACCAACAATTCCATTAAG***/GACTCGAGGGCGCCGGAA |
|  | TB181 | CGAGTC/***CTTAATGGAATTGTTGGTTCA***/ATGGCGGAGGACACGGAG |
| pBY-JK2.P12/KoA_eIF4E_T120S (NIb/CP) | TB244 | CAACTGAT/GcT/CCATTTACCGCCATTGG |
|  | TB243 | GTAAATGG/AgC/ATCAGTTGTGGCAAAGG |
| pBY-JK2.P12/KoA_eIF4E_N160D(NIb/CP) | TB286 | CTCTTTCCTG/GTc/CTTACGCACGCTGACGAC |
|  | TB285 | GTGCGTAAG/gAC/CAGGAAAGAGTAGCTATCT |
| pBY-JK2.P12/KoA_eIF4E_Q161K(NIb/CP) | TB288 | CTCTTTC/CTt/GTTCTTACGCACGCTGACGAC |
|  | TB287 | GTGCGTAAGAAC/aAG/GAAAGAGTAGCTATCT |
| pBY-JK2.P12/*rym5*_eIF4E_D160N(NIb/CP) | TB288 ^b^ |  |
|  | TB287 ^b^ |  |
| pBY-JK2.P12/*rym5*_eIF4E_K161Q(NIb/CP) | TB286 ^b^ |  |
|  | TB285 ^b^ |  |

^a^ Plain letters, derived from viral genome or *eIF4E* gene; framed letters, restriction enzyme sequences; bold-face letters, T7 promoter sequence; italic bold-face letters, GFP-coding sequence; lower-case letters, substituted nucleotides; underlined letters, substituted codons; underlined italic bold-face letters, (NIb/CP) or (P1/P2) cleavage site sequences.

^b^ These primers have been shown in the above mentioned constructions.
